# Association between police work stress and life satisfaction: the chain mediating roles of perceived social support and sleep disturbances

**DOI:** 10.3389/fpsyg.2026.1938729

**Published:** 2026-09-17

**Authors:** Jiwen Qiu, Xianghui Lai, Jue Deng, Kunyan Wang

**Affiliations:** 1 Fujian Police College, Fuzhou, China; 2 Department of Basic Courses, Fujian Police College, Fuzhou, China; 3 Cognitive Neuroscience and Abnormal Psychology Laboratory, Department of Penalty Execution, Fujian Police College, Fuzhou, China

**Keywords:** chain mediation, life satisfaction, perceived social support, police work stress, sleep disturbances

## Abstract

**Objective:**

Policing is characterized by persistent and pervasive stressors that systematically undermine officers’ overall life evaluations and well-being. Although studies have confirmed a direct link between police work stress and life satisfaction, the sequential pathways through which perceived social support and sleep impairment operate in this relationship remain significantly underexplored. This study investigates the direct relationship between police work stress and life satisfaction, and the independent and chained mediating effects of perceived social support and sleep disturbances, among Chinese active-duty police officers.

**Methods:**

Through a cross-sectional survey, we collected data from 803 frontline police in southeast China using four standardized scales: the Police Stress Scale, Satisfaction with Life Scale, Social Support Rating Scale, and Pittsburgh Sleep Quality Index. Statistical software (SPSS version 26.0 and AMOS version 26.0) was used for correlation analysis, structural equation modeling, and bootstrap mediation analysis. Demographic covariates were controlled to verify model robustness.

**Results:**

All variables showed significant correlations (*p* < 0.001). Police work stress directly and negatively predicted life satisfaction (*B* = −0.167, *p* = 0.001), and perceived social support and sleep disturbances separately mediated the focal association. A significant serial chained path was validated: police work stress → perceived social support → sleep disturbances → life satisfaction (B = −0.015, *p* = 0.003). All pathways remained stable after covariate adjustment.

**Conclusion:**

There are significant associations between police work stress and police life satisfaction through direct, single-mediating, and serial chained correlational pathways. Police work stress is correlated with lower perceived social support, poorer sleep quality, and reduced life satisfaction among officers. Police departments should use integrated interventions to strengthen multi-level social support and its utilization, and provide sleep health programs to improve officers’ life satisfaction.

## 1 Introduction

Police work stress is defined as the psychological strain and burden arising from inherent occupational demands, including law enforcement risks, organizational management, and public interactions (Chen and Hu, 2012; Trépanier et al., 2026). Because police officers routinely operate under high levels of such stress (Ugwu and Idemudia, 2025; Queirós et al., 2020), these chronic exposures exert deleterious effects on both their professional efficacy and their holistic physical and mental health (Anders et al., 2022; Nelson and Smith, 2023; Violanti et al., 2025). Existing research has validated that chronic police occupational stress predicts poorer life satisfaction (Purba and Demou, 2019; Alexopoulos et al., 2014), yet few studies unpack its underlying psychological pathways. Clarifying the intermediary mechanisms linking occupational stress to life satisfaction can provide empirical evidence for developing targeted stress interventions and well-being promotion strategies for frontline police officers (Wu et al., 2025).

### 1.1 Literature review

Diminished life satisfaction is a critical outcome in the literature on police stress (Lambert et al., 2021). Conceptually, life satisfaction reflects an individual’s overall appraisal of quality of life, with higher scores indicating greater subjective well-being (Diener et al., 1985; Pavot and Diener, 1993). Life satisfaction serves as a robust indicator of police officers’ overall life status and is closely associated with occupational stress (Liu et al., 2019).

Police work stress appears related to various factors, including the working environment, organizational factors, and social resources (Anders et al., 2022; Bezie et al., 2024; Hansson and Padyab, 2023). Within the context of chronic occupational pressure, police officers often report significant deficits in perceived social support across emotional, instrumental, and organizational dimensions (Cheung and Li, 2023).

Perceived social support refers to individuals’ subjective perception and utilization of assistance from family, friends, colleagues, and institutions (Liu et al., 2008; Xiao, 1994). A few studies have confirmed that perceived social support is related to both police work stress and mental health (Yang and Tang, 2026; Sun et al., 2023; Hansson and Padyab, 2023). The stress-buffering hypothesis lays the theoretical foundation for its mediating role between police work stress and life satisfaction (Alvarado-García et al., 2026; Lam, 2024; Cohen and Wills, 1985). This theory identifies two sequential protective functions of social support (Davis et al., 2025; Cohen and Wills, 1985): primary appraisal buffering and secondary recovery buffering. In primary appraisal, multidimensional support reframes officers’ perceptions of occupational hazards, overtime, and public conflicts to reduce stress severity. In secondary buffering, supportive resources relieve post-stress distress, prevent work strain from spilling over into personal domains, and safeguard life satisfaction (Gillman et al., 2023). Acting as a “protective shield,” social support mitigates job pressure’s harmful impacts on mental well-being (Cohen and Wills, 1985; Gillman et al., 2023). Restricted by the rigid hierarchical system within police forces and frequent shift work, officers often face insufficient peer, supervisory and familial support (Hansson and Padyab, 2023; Rauschmayr et al., 2023; Wolter et al., 2019). Support deficits hinder stress recovery, weaken coping ability, trigger negative emotions, and eventually lower life satisfaction (Hansson and Padyab, 2023; McCanlies et al., 2018; Padhy et al., 2023). Accordingly, perceived social support may serve as a mediator between police work stress and life satisfaction.

In addition, robust evidence links the demanding features of police work, including irregular shift patterns, constant hypervigilance, and exposure to critical incidents, to poor sleep quality and more sleep complaints among officers (Chen et al., 2025; Galanis et al., 2021; Chopko et al., 2021; Garbarino et al., 2019). Åkerstedt’s (2006) sleep-stress model theoretically explains the effect pathway: chronic occupational stress activates sustained physiological and cognitive arousal, which directly disturbs sleep architecture and undermines sleep maintenance. Consistent with this framework, elevated police work stress precipitates sleep disruption, driven primarily by persistent psychological arousal, cumulative trauma, and erratic schedules (Åkerstedt, 2006; Linton et al., 2015). Crucially, such disruptions impede psychophysiological recovery by intensifying fatigue, burnout, and somatic symptoms, reducing officers’ positive evaluations of their overall life situation (Kyle et al., 2010; Rajaratnam et al., 2011; Su and He, 2023). These deleterious effects extend beyond the workplace, compromising both occupational outcomes (i.e., job performance and safety; Ye et al., 2023) and general well-being, manifesting as diminished job satisfaction and impaired daily functioning (Baker et al., 2020). Therefore, sleep disturbances may serve as another mediator between police work stress and life satisfaction.

The conservation of resources (COR) theory (Hobfoll, 1989) further provides a compelling framework for this study, suggesting that sustained occupational strain gradually erodes an individual’s pool of emotional, cognitive, and interpersonal resources. From this theoretical perspective, adequate social support is a critical buffer, enabling adaptive appraisal of stressors, provision of emotional solace, and restoration of psychological security. Conversely, deficient social support signifies not only a scarcity in aid from colleagues, supervisors, and family, but also impaired capacity for psychophysiological recovery. Without sufficient external resource replenishment from social connections, the lingering stress arousal triggered by policing duties cannot be effectively downregulated. This impairment heightens psychological arousal during off-duty hours, elevating the risk of sleep disturbances (de Grey et al., 2018). Accordingly, for police officers, work stress manifests initially as depleted perceived support, which exacerbates sleep impairment, culminating in maladaptive symptoms (Angehrn et al., 2022; Chen et al., 2025). In this resource loss spiral, poor sleep in turn consumes remaining psychological reserves and cuts off the path to effective resource recuperation. Persistent sleep disruption further intensifies fatigue, emotional dysregulation, and adverse health perceptions, ultimately eroding positive life appraisals and overall life satisfaction (Baker et al., 2020; Banerjee and Boro, 2022). Taken together, the interplay between perceived social support and sleep disturbances (Gordon et al., 2021) indicates a potentially chained mediation mechanism between police stress and life satisfaction.

### 1.2 Research gaps

Despite consistent evidence confirming the negative effect of police occupational stress on life satisfaction (Baker et al., 2020; Liu et al., 2019; Alexopoulos et al., 2014), and the independent mediating functions of perceived social support and sleep disturbances (Yang and Tang, 2026; Birhan et al., 2025), notable research gaps persist in current literature. Most existing studies merely explore direct associations or separate parallel mediating pathways and fail to integrate social support and sleep health into a unified theoretical system. Guided by the Stress-buffering Hypothesis (Davis et al., 2025; Cohen and Wills, 1985), COR theory (Hobfoll, 1989), and Åkerstedt’s sleep-stress model (Åkerstedt, 2006), occupational stress triggers a cascading resource loss spiral: it depletes social supportive resources first, induces physiological arousal and sleep impairments, and ultimately reduces life satisfaction. However, this sequential chained mechanism remains empirically underexamined among frontline police officers.

Furthermore, relevant empirical evidence is scarce for Chinese frontline police samples. Characterized by rigid hierarchical administration, high-risk work scenarios, and frequent shift work, Chinese policing possesses unique occupational features, which may shape distinct stress transmission pathways. China’s distinct social, cultural, and operational contexts—such as severe understaffing relative to its massive population, strict gun control regulations, and a hierarchical organizational structure in the police force that demands unquestioned obedience—create a highly stressful work environment for police officers (Lu et al., 2015; Wang et al., 2014). Accordingly, this study addresses the above gaps by establishing a serial mediation model to clarify the sequential mediating roles of perceived social support and sleep disturbances in the stress–life satisfaction linkage.

### 1.3 Hypothesized research model

In summary, this study proposes a serial mediation model (Figure 1) to elucidate the relationships among police work stress, life satisfaction, perceived social support, and sleep disturbances. A sequential pathway is hypothesized, wherein occupational stress is associated with lower perceived social support, which, in turn, predicts greater sleep disturbances, ultimately culminating in lower life satisfaction. This framework delineates the latent psychological mechanisms linking work stress to subjective well-being. Practically, it offers theoretical guidance for mitigating occupational stress and enhancing life satisfaction among police officers, while also potentially informing comprehensive pre- and in-service mental health education programs.

**Figure 1:**
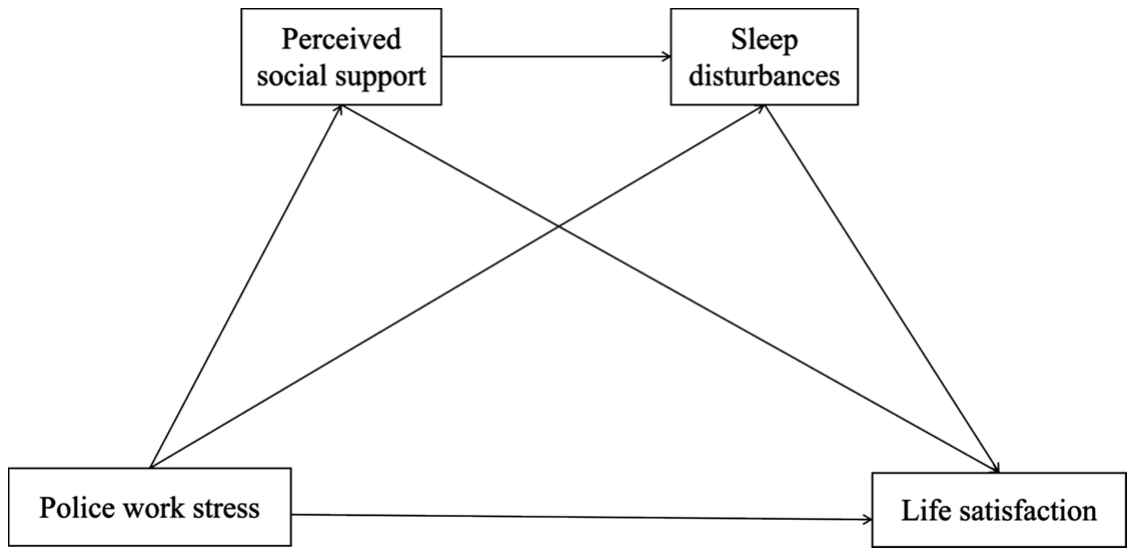
Hypothesized study model.

## 2 Materials and methods

### 2.1 Participants

Data were collected from active-duty police officers stationed in southeastern Chinese mainland. Prior to survey administration, the research protocol obtained operational clearance for field implementation from police staff responsible for officers’ mental-health services. Recruitment information was then disseminated to all tenured police officers across three jurisdictions with the assistance of police staff overseeing administrative affairs and officers’ mental health. Questionnaires, together with all measurement scales, were converted into web-based links accessible via mobile phones or computers. Each police officer independently decided whether to participate after understanding the questionnaire content and study objectives. Participants were required to provide informed consent prior to gaining access to the survey link. All police officers participating in the study completed the questionnaire online via this link. Participants consisted of serving police officers from diverse functional divisions covering multiple police units: criminal investigation, public security administration, traffic management, prison and correctional services, exit-entry administration, and other relevant departments. All participants received explicit assurance that participation was voluntary and would not impact their job standing, and that they had the right to withdraw at any stage without negative effect. Participants were further assured that any concerns or complaints regarding the study would be addressed confidentially. To safeguard privacy, no identifiable data were accessible to third parties, including the participants’ units. The dataset relied exclusively on self-reported measures. The research protocol received formal ethical approval from the Institutional Review Board of Fujian Police College (approval ID: FPCER-202601-004). All investigative procedures were rigorously carried out according to the principles of the 1964 Declaration of Helsinki and its subsequent amendments.

In total, 824 active-duty police officers completed the questionnaire via the link. After data screening, 21 questionnaires were excluded owing to excessive missing values (exceeding 10% of all responses) or patterned responding, for a final valid sample of 803 officers (effective response rate = 97.45%). Table 1 presents the participants’ demographic characteristics. The sample comprised individuals aged 20–63 years (mean [M] = 39.44, standard deviation [SD] = 10.24), with professional tenure ranging from < 1 year to 43 years (M = 16.16, SD = 11.20). It should be clarified that the extreme-age participants constituted a very small proportion of the sample (three aged 20 and one aged 63), and their active-duty status was verified with the local public security personnel administrators. The 20-year-old officers were newly recruited graduates of a five-year vocational program holding a full-time associate degree, while the 63-year-old officer was retained under a local provision extending service for officers with specialized skills or in core positions, in line with China’s phased retirement-age postponement (Xinhua News Agency, 2024).

**Table 1:** Participant demographics (*N* = 803).

Variable	Category	Frequency
Gender	Male	684 (85.2%)
	Female	119 (14.8%)
Workplace location	Urban	612 (76.2%)
	Rural	191 (23.8%)
Educational level	Senior high	27 (3.4%)
	College diploma	188 (23.4%)
	Bachelor’s degree	566 (70.5%)
	Master’s degree	20 (2.5%)
	Doctoral degree	2 (0.2%)
Marital status	Unmarried	169 (21.0%)
	First marriage	552 (68.7%)
	Remarried	33 (4.1%)
	Divorced	47 (5.9%)
	Widowed	2 (0.2%)
Number of children	No children	218 (27.1%)
	1 child	332 (41.3%)
	2 children	238 (29.6%)
	≥3 children	15 (1.9%)

### 2.2 Measures

#### 2.2.1 Police stress scale (PSS)

To quantify occupational strain, we used the PSS. The PSS was originally developed and validated by Chen and Hu (2012) specifically for Chinese police samples, demonstrating strong psychometric properties (Wu, 2022; Zheng, 2025). This 50-item instrument captures six distinct facets of police stress: work tasks (e.g., heavy caseloads), social life (e.g., work–family conflict), power motivation (e.g., perceived unfairness), personal ability (e.g., skill deficits), negative emotions (e.g., safety anxieties), and organizational management (e.g., excessive bureaucracy).

Item scoring follows a two-stage process: respondents first indicate the occurrence of a stressor (Yes/No), and then rate its perceived impact on a 5-point Likert scale ranging from 1 (No impact) to 5 (Extremely heavy impact). Specifically, if the stressor described in an item does not occur (participants select “no”), no impact rating is required for that item, and the item receives no score (0 point). If a stressor described in an item occurs (participants select “yes” for the “occurred” response option), participants provide the corresponding impact rating (From 1 to 5). Notably, this is a two-stage scoring system, not a binary either-or scoring method (Chen and Hu, 2012). Higher scores on the PSS correspond to greater levels of stress.

In this study, the scale exhibited exceptional internal consistency, with a Cronbach’s α of 0.988 and a McDonald’s ω of 0.991. The internal consistency of the six dimensions of PSS was also calculated: for work tasks, Cronbach’s α = 0.963 and McDonald’s ω = 0.964; for social life, Cronbach’s α = 0.955 and McDonald’s ω = 0.956; for power motivation, Cronbach’s α = 0.963 and McDonald’s ω = 0.964; for personal ability, Cronbach’s α = 0.937 and McDonald’s ω = 0.940; for negative emotions, Cronbach’s α = 0.936 and McDonald’s ω = 0.937; for organizational management, Cronbach’s α = 0.957 and McDonald’s ω = 0.957. All sub-scales exhibited excellent internal consistency.

#### 2.2.2 Satisfaction with life scale (SWLS)

The SWLS (Diener et al., 1985), the applicability and validity of which have been extensively confirmed in Chinese populations, was administered to measure participants’ global life satisfaction (Xiong and Xu, 2009; Song et al., 2014). The SWLS comprises five items (e.g., “In most ways, my life is close to my ideal”) rated on a 7-point Likert scale ranging from 1 (Strongly disagree) to 7 (Strongly agree). The scale demonstrated excellent internal consistency in this study (Cronbach’s α = 0.947; McDonald’s ω = 0.949).

#### 2.2.3 Social support rating scale (SSRS)

We assessed perceived social support using the SSRS, originally developed by Xiao (1994) and extensively validated in Chinese samples (Liu et al., 2008). This 10-item instrument taps into three subdomains: subjective support (4 items, reflecting perceived respect and understanding), objective support (3 items, measuring tangible aid and network contacts), and support utilization (3 items, assessing proactive help-seeking behaviors).

The SSRS adopts a mixed scoring format combining fixed 4-point Likert scoring and source-count scoring for multi-select items: (1) Four-point Likert-scored items (1 = None/Rarely to 4 = Full / Frequently): Items 1–4 and Items 8–10 each contain a single response stem with four ordered options, assigned 1 to 4 points directly based on selection. Item 5 includes five subdomains of family support; each sub-item is rated 1–4 from “no support” to “full support,” and the sum of its five subscores forms the total score of item 5. (2) Source-count scoring items (Items 6 and 7): These two items ask participants to select all sources that provided material aid (Item 6) or emotional comfort (Item 7) during stressful events. If respondents select “no source available,” the item is scored 0; for respondents endorsing any of the listed support sources, one point is awarded for each selected source.

The SSRS demonstrated acceptable internal consistency in this sample (Cronbach’s α = 0.719; McDonald’s ω = 0.756), exceeding the conventional threshold of 0.70 (Tavakol and Dennick, 2011).

#### 2.2.4 Pittsburgh sleep quality index (PSQI)

To evaluate sleep quality, we used the Pittsburgh Sleep Quality Index (PSQI; Buysse et al., 1989), the Chinese version of which has been rigorously validated for local populations (Liu et al., 1996). This 19-item instrument yields seven component scores: subjective sleep quality, sleep latency, duration, habitual efficiency, disturbances, hypnotic medication use, and daytime dysfunction. Each component is rated on a 0–3 ordinal scale, with a global sum score of 0–21. Higher totals denote more pronounced sleep impairment. Within our sample, the PSQI exhibited robust reliability, evidenced by a Cronbach’s α of 0.788 and a McDonald’s ω of 0.792, both comfortably exceeding the recommended 0.70 benchmark (Tavakol and Dennick, 2011). These values are largely consistent with prior psychometric evaluations across diverse cohorts (e.g., Buysse et al., 1989; Zhong et al., 2012; Dou et al., 2024).

### 2.3 Statistical analysis

All statistical procedures were carried out using SPSS 26.0 (IBM Corp., Armonk, NY, USA) and Amos 26.0 (IBM Corp.). In the preliminary phase, we computed descriptive statistics, internal consistency (both Cronbach’s α and McDonald’s ω), bivariate correlations, and collinearity diagnostics using SPSS with a third-party plugin to confirm data appropriateness. Subsequently, we used Amos 26.0 to construct a structural equation model (SEM) testing our hypothesized serial mediation; specifically, whether perceived social support and sleep disturbances sequentially mediated the effect of police work stress on life satisfaction.

### 2.4 Common method bias (CMB)

Prior to data collection, several procedural strategies were implemented to minimize potential common-method bias, including covering multiple police specialties, guaranteeing full participant anonymity in survey instructions, emphasizing the absence of right or wrong answers to mitigate socially desirable responding, and informing participants that they could withdraw from the survey at any time without any negative consequences. We further performed statistical tests to evaluate the presence of common-method bias. First, CMB was assessed using the Harman single-factor test. Unrotated principal component analysis extracted 17 factors with eigenvalues > 1.0, with the first factor accounting for 35.33% of the total variance—below the critical threshold of 40% (Zhou and Long, 2004).

To further assess potential CMB, confirmatory factor analysis (CFA) was then performed. We compared the model fit of a single-factor model with that of a four-factor model. In these models, the observed variables were the sub-scale (sub-dimension) scores of each individual scale, except for the SWLS. Because life satisfaction represents a unidimensional latent construct, sub-dimension scores cannot be generated for this measure. Per CFA model-identification requirements in AMOS, each latent construct needs a minimum of three observed indicators to yield an identifiable measurement model. Accordingly, we entered the five original items of the SWLS as observed indicators in the CFA models for common-method-bias assessment.

The single-factor model demonstrated a poor fit to the data (χ^2^/degrees of freedom [df] = 29.811, CFI = 0.585, TLI = 0.538, RMSEA = 0.190, SRMR = 0.166). By contrast, the hypothesized four-factor measurement model had a significantly improved fit (χ^2^/df = 4.884, CFI = 0.946, TLI = 0.938, RMSEA = 0.070, SRMR = 0.047). A chi-square difference test confirmed that the four-factor model was statistically superior to the single-factor model (Δχ^2^ = 4740.414, Δdf = 6, *p* < 0.001). Collectively, these results indicate the absence of severe CMB.

More detailed information about the CMB test is provided in the Supplementary Figures S1, S2, Supplementary Table S1. Of note, in these two comparison models, the PSQI was represented by its sub-dimension scores, whereas the PSQI was included as a total score in all subsequent analyses.

## 3 Results

### 3.1 Descriptive statistics, composite reliability (CR), discriminant validity, and correlations

Descriptive statistics and normality assessments were conducted for the four primary constructs—work stress, life satisfaction, sleep disturbances, and perceived social support—along with their sub-dimensions (Table 2). Skewness values ranged from −0.455 to 1.547, and kurtosis values from −1.062 to 1.425. Both indices were well within the acceptable thresholds of |skewness| < 3 and |kurtosis| < 7 (Kline, 2011), indicating no significant non-normality. Consequently, the data met the assumptions for subsequent parametric analyses and structural equation modeling.

**Table 2:** Descriptive statistics, skewness, and kurtosis of the study variables (*N* = 803).

Variables	*M*	*SD*	Skewness	Kurtosis
Police work stress	1.787	1.194	0.388	−0.479
Work tasks	2.347	1.434	0.043	−0.907
Social life	1.840	1.233	0.407	−0.399
Power motivation	1.507	1.343	0.684	−0.387
Personal ability	1.457	1.172	0.659	−0.011
Negative emotions	1.609	1.328	0.678	−0.136
Organizational management	1.761	1.327	0.452	−0.511
Life satisfaction	3.756	1.557	0.123	−0.412
Pittsburgh Sleep Quality Index	8.600	3.937	0.302	−0.339
Subjective sleep quality	1.590	0.798	0.013	−0.487
Latency	1.717	0.992	−0.164	−1.062
Duration	1.385	0.739	0.735	0.124
Efficiency	0.550	0.874	1.547	1.425
Disturbances	1.299	0.862	0.285	−0.530
Use of hypnotic medication	0.179	0.595	3.582	12.365
Daytime dysfunction	1.879	1.000	−0.455	−0.893
Perceived social support	3.627	0.960	−0.027	−0.285
Subjective support	5.407	1.414	−0.245	−0.630
Objective support	2.587	1.149	0.448	0.399
Utilization of support	2.295	0.768	0.380	−0.427

At the sub-dimension level, all components satisfied the normality assumptions except for “use of hypnotic medication” (PSQI6). This component exhibited significant positive skewness (3.582) and leptokurtosis (12.365), indicating a pronounced deviation from normality. Therefore, a rank-based normal transformation was applied to the total PSQI score and its seven constituent factor scores. Post-transformation, the skewness of PSQI6 decreased to 2.835, and kurtosis to 6.474, bringing the data within acceptable limits for parametric analysis.

Table 3 shows that the CR values for work stress, life satisfaction, and perceived social support all exceeded the recommended threshold of 0.70, with average variance extracted (AVE) values surpassing the 0.50 benchmark (Fornell and Larcker, 1981). These results confirm adequate internal consistency and convergent validity for these constructs. Regarding sleep disturbances (PSQI), the CR was 0.792, indicating robust internal consistency (Bagozzi and Yi, 2012). However, the AVE value was 0.370, below the conventional 0.50 cutoff. This result suggests relatively weaker convergent validity for the PSQI, likely because of the scale’s multidimensional nature or the presence of skewed items (e.g., hypnotic use) (Buysse et al., 1989; Cole et al., 2006; Liu et al., 2021). Therefore, the PSQI was integrated into the structural model as a composite factor score rather than using individual item-level data. We further assessed discriminant validity using the Fornell–Larcker criterion (Fornell and Larcker, 1981). The square root of the AVE for each latent construct was compared against all inter-construct correlation coefficients; in all cases, the AVE square root surpassed the corresponding highest correlation, providing empirical support for adequate discriminant differentiation among all study dimensions. The PSQI demonstrated acceptable discriminant validity, with an AVE square root of 0.608 exceeding its correlations with other latent variables. It should be noted that in the confirmatory factor-analysis reported in Table 3, life satisfaction was specified as a latent construct using five original SWLS items as indicators for psychometric validation. In the subsequent primary serial-mediation structural model analysis, life satisfaction was included as a composite observed variable based on item-parceling of the five SWLS items, as the SWLS is a unidimensional construct.

**Table 3:** Factor loadings, CR, AVE, and Pearson correlation matrix.

Dimension	Standardized factor loading	CR	AVE	1	2	3	4
1 Police work stress	[0.839, 0.949]	0.962	0.809	0.899			
2 Life satisfaction	[0.775, 0.932]	0.949	0.787	−0.383^***^	0.887		
3 Pittsburgh Sleep Quality Index	[0.317, 0.794]	0.792	0.370	0.565^***^	−0.362^***^	0.608	
4 Perceived social support	[0.701, 0.757]	0.766	0.521	−0.439^***^	0.422^***^	−0.383^***^	0.722

The diagonal values represent the square root of AVE, whereas values below the diagonal represent the Pearson correlation coefficients between dimensions. ^***^p < 0.001. CR, composite reliability; AVE, average variance extracted.

To further assess discriminant validity at the dimensional level, we adopted the Heterotrait−Monotrait ratio (HTMT) criterion. According to Henseler et al. (2015), an HTMT value < 0.85 indicates satisfactory discriminant validity across constructs, whereas values < 0.90 represent an acceptable threshold under more relaxed criteria. All HTMT estimates for the four dimensions fell below the strict cutoff of 0.85 (range: 0.402–0.636), confirming robust discriminant validity between distinct dimensions. These findings demonstrate that the four core constructs (work stress, life satisfaction, sleep disturbances, and perceived social support) are statistically distinguishable, with no severe cross-dimensional collinearity or construct overlap, corroborating the soundness of the measurement model.

### 3.2 Model fit for structural equation modeling

A serial multiple mediation model was constructed via AMOS 26.0 to test the hypothesized chain effect. Police work stress was treated as the independent variable, life satisfaction served as the outcome variable, while perceived social support and sleep disturbances were set as sequential mediating variables in the proposed pathway. Table 4 presents the goodness-of-fit statistics for the structural model. The model yielded a chi-square value of 439.087 with 85 df, producing a χ^2^/df ratio of 5.166. This ratio marginally exceeds the conventional cutoff of 5; thus, this index does not meet the ideal threshold. Nevertheless, because the χ^2^ test is highly sensitive to trivial model misspecifications when large sample sizes are involved, model fit must be evaluated holistically alongside supplementary fit metrics (Bentler and Bonett, 1980).

**Table 4:** Goodness-of-fit index of the structural model.

Fit indices	Criteria	Research model fit
*χ^2^/df*	1 < *χ^2^*/*df* < 5	5.166
RMSEA	<0.08	0.072
SRMR	<0.08	0.024
RMR	<0.08	0.046
GFI	>0.90	0.935
AGFI	>0.90	0.908
NFI	>0.90	0.962
RFI	>0.90	0.953
IFI	>0.90	0.969
TLI	>0.90	0.961
CFI	>0.90	0.969

χ^2^, chi-square; df, degrees of freedom; RMSEA, root mean square error of approximation; SRMR, standardized root mean square residual; RMR, root mean square residual; TLI, Tucker–Lewis index; NFI, normed fit index; CFI, comparative fit index; GFI, goodness-of-fit index; AGFI, adjusted goodness-of-fit index; IFI, incremental fit index; RFI, relative fit index.

As shown in Table 4, in terms of absolute fit indices, the structural equation model constructed in this study exhibited acceptable overall fit, rendering it suitable for subsequent path coefficient calculation and serial mediation effect analysis (MacCallum et al., 1996; Molwus et al., 2013; Sahoo, 2019).

### 3.3 Direct and indirect effect analyses

Table 5 reports the direct path estimates derived from the SEM. All hypothesized paths were statistically significant at *p* < 0.01. Figure 2 illustrates the standardized direct path coefficients of the structural model. Police work stress was significantly and negatively associated with life satisfaction (B = −0.167, *p* = 0.001). In addition, police work stress was significantly and negatively associated with perceived social support (B = −0.446, *p* < 0.001) but was significantly and positively associated with sleep disturbances (B = 0.363, *p* < 0.001). In turn, perceived social support was significantly and negatively associated with sleep disturbances (B = −0.200, *p* < 0.001) and significantly and positively associated with life satisfaction (B = 0.526, *p* < 0.001). Furthermore, sleep disturbances were significantly and negatively associated with life satisfaction (B = −0.171, *p* = 0.005). Collectively, these findings indicate that police work stress and sleep disturbances were associated with lower life satisfaction, whereas perceived social support was associated with higher life satisfaction.

**Table 5:** Estimates of direct model pathways.

Path	B	β	SE	CR	*p*
Police work stress → perceived social support	−0.446	−0.517	0.035	−12.885	<0.001
Perceived social support → sleep disturbances	−0.200	−0.217	0.038	−5.216	<0.001
Police work stress → sleep disturbances	0.363	0.456	0.029	12.408	<0.001
Perceived social support → life satisfaction	0.526	0.378	0.067	7.810	<0.001
Police work stress → life satisfaction	−0.167	−0.139	0.052	−3.209	0.001
Sleep disturbances → life satisfaction	−0.171	−0.114	0.061	−2.827	0.005

B, unstandardized coefficient; β, standardized coefficient; SE, standard error; CR, critical ratio.

**Figure 2:**
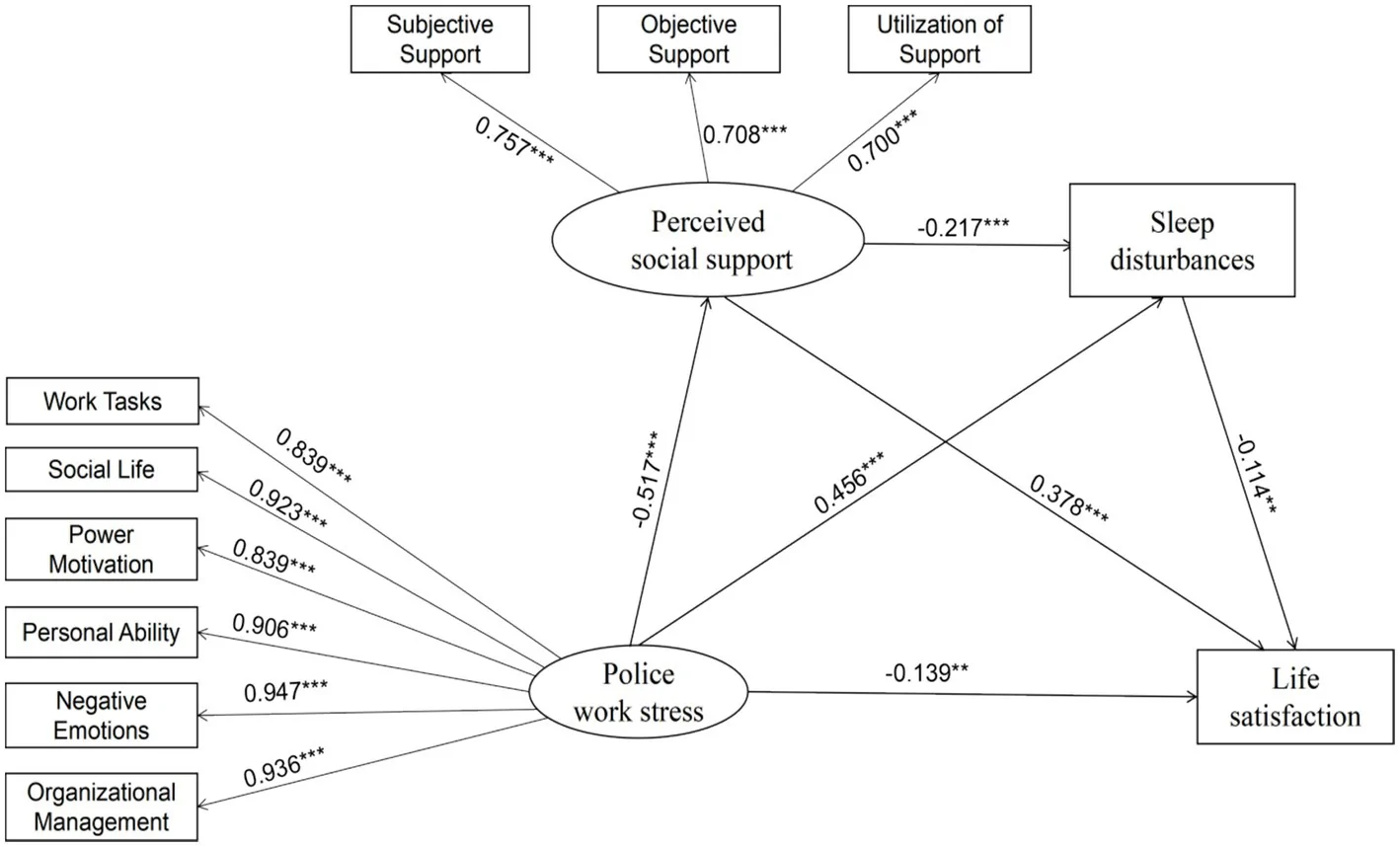
Standardized model path coefficients. The numbers attached to single-headed arrows reflect the standardized path coefficients. ^**^p < 0.01; ^***^p < 0.001.

Table 6 presents the indirect, direct, and total effect estimates derived from the SEM. First, perceived social support had a significant negative indirect mediating effect on the relationship between police work stress and life satisfaction (B = −0.234, *p* < 0.001). Second, sleep disturbances exerted a significant negative mediating effect on the relationship between police work stress and life satisfaction (B = −0.062, *p* = 0.004). Police work stress exerted a significant negative serial indirect effect on life satisfaction via the sequential pathway of perceived social support and sleep disturbances (B = −0.015, *p* = 0.003). The total indirect effect was negative and statistically significant (B = −0.312, *p* < 0.001). In addition, the direct effect of police work stress on life satisfaction was negative and significant (B = −0.167, *p* = 0.004), yielding a significant negative total effect (B = −0.479, *p* < 0.001). Collectively, these results indicate that police work stress was negatively associated with life satisfaction through both a direct pathway and three specific indirect pathways involving perceived social support and sleep disturbances.

**Table 6:** Estimated indirect model pathways.

Path	B	SE	Z	BC 95% CI	*p*
Police work stress → perceived social support → life satisfaction	−0.234	0.039	−6.000	[−0.314, −0.163]	<0.001
Police work stress → sleep disturbances → life satisfaction	−0.062	0.023	−2.696	[−0.109, −0.019]	0.004
Police work stress → perceived social support → sleep disturbances → life satisfaction	−0.015	0.006	−2.500	[−0.030, −0.005]	0.003
Aggregate indirect effect	−0.312	0.044	−7.091	[−0.403, −0.232]	<0.001
Direct effect	−0.167	0.062	−2.694	[−0.291, −0.047]	0.004
Total effect	−0.479	0.051	−9.392	[−0.581, −0.378]	<0.001

Maximum likelihood estimation method with 5,000 bootstrap values was used. B, unstandardized coefficient; SE, standard error; BC 95% CI, bias-corrected 95% confidence interval.

### 3.4 Robustness test

To verify the robustness of the core findings, demographic covariates (gender, age, police tenure, workplace location, and marital status) were incorporated into the SEM as control variables. In the covariate-adjusted robustness model, the measurement portion remained identical to the original primary SEM with no modifications to factor indicators, factor loadings, or measurement-error specifications. The goodness-of-fit statistics for this adjusted model were generally acceptable: χ^2^ = 879.280, df = 154, χ^2^/df = 5.710, CFI = 0.948, TLI = 0.935, RMSEA = 0.077, and SRMR = 0.075. Cross-covariances between the control variables and the indicators of the hypothesized structural model were constrained by the model’s structural specification and thus did not add new free parameters. Although variance–covariance terms among the exogenous control variables consumed additional degrees of freedom, these constrained cross-covariances provided over-identifying information for the model, yielding a net increase in degrees of freedom (Δdf = 69).

Table 7 reports the path estimates for the structural model with control variables. Table 8 reports the path coefficient estimates of the predictive effects of demographic controls on perceived social support, sleep disturbances, and life satisfaction. Table 9 reports the indirect, direct, and total effects in the covariate-adjusted model. Police work stress retained a significant negative predictive effect on perceived social support (B = −0.462, *p* < 0.001) and a significant positive predictive effect on sleep disturbances (B = 0.359, *p* < 0.001). In turn, perceived social support significantly and negatively predicted sleep disturbances (B = −0.234, *p* < 0.001) and significantly and positively predicted life satisfaction (B = 0.517, *p* < 0.001). Sleep disturbances also had a significant negative direct effect on life satisfaction (B = −0.167, *p* = 0.007). Finally, the direct path from police work stress to life satisfaction remained statistically significant and negative (B = −0.154, *p* = 0.005). Collectively, these findings demonstrate that after accounting for gender, age, police tenure, workplace location, and marital status, the primary structural relationships among police work stress, perceived social support, sleep disturbances, and life satisfaction were largely unchanged, confirming the stability of the core path results. The covariate-adjusted model is provided in the Supplementary Figure S3.

**Table 7:** Model path estimates with control variables included.

Path	B	*β*	SE	CR	*p*
Police work stress → perceived social support	−0.462	−0.510	0.037	−12.474	<0.001
Perceived social support → sleep disturbances	−0.234	−0.256	0.039	−5.939	<0.001
Police work stress → sleep disturbances	0.359	0.435	0.032	11.405	<0.001
Perceived social support → life satisfaction	0.517	0.379	0.070	7.362	<0.001
Police work stress → life satisfaction	−0.154	−0.125	0.055	−2.780	0.005
Sleep disturbances → life satisfaction	−0.167	−0.111	0.062	−2.680	0.007

The model controlled for gender, age, police tenure, workplace location, and marital status. B, unstandardized coefficient. β, standardized coefficient; SE, standard error; CR, critical ratio.

**Table 8:** Estimates of paths between control and endogenous variables.

Control variable path	*β*	*p*
Female → perceived social support	0.021	0.550
Age → perceived social support	0.041	0.755
Police tenure → perceived social support	−0.016	0.903
Rural work location → perceived social support	−0.025	0.495
Currently married → perceived social support	0.273	<0.001
Female → sleep disturbances	0.050	0.081
Age → sleep disturbances	0.133	0.205
Police tenure → sleep disturbances	0.034	0.740
Rural work location → sleep disturbances	0.011	0.710
Currently married → sleep disturbances	0.042	0.216
Female → life satisfaction	0.076	0.019
Age → life satisfaction	−0.086	0.470
Police tenure → life satisfaction	0.044	0.706
Rural work location → life satisfaction	−0.020	0.538
Currently married → life satisfaction	0.011	0.766

β represents the standardized path coefficient.

**Table 9:** Estimated indirect, direct, and total effects in the covariate-adjusted model.

Path	B	SE	Z	BC 95% CI	*p*
Police work stress → perceived social support → life satisfaction	−0.239	0.043	−5.558	[−0.327, −0.157]	<0.001
Police work stress → sleep disturbances → life satisfaction	−0.060	0.023	−2.609	[−0.108, −0.018]	0.008
Police work stress → perceived social support → sleep disturbances → life satisfaction	−0.018	0.007	−2.571	[−0.035, −0.006]	0.006
Aggregate indirect effect	−0.317	0.047	−6.745	[−0.415, −0.230]	<0.001
Direct effect	−0.154	0.066	−2.333	[−0.286, −0.025]	0.018
Total effect	−0.470	0.055	−8.545	[−0.578, −0.363]	<0.001

Maximum likelihood estimation method with 5,000 bootstrap values was used. B, unstandardized coefficient; SE, standard error; BC 95% CI, bias-corrected 95% confidence interval.

## 4 Discussion

Substantial literature has documented a link between police work stress and reduced life satisfaction or compromised subjective well-being (Alexopoulos et al., 2014; Angehrn et al., 2022; Chen et al., 2025; Lambert et al., 2021; Ryu et al., 2020). Nevertheless, the specific sequential mechanisms that might explain how work stress translates into reduced life satisfaction, particularly through a combined effect of lower perceived social support and sleep problems, remain insufficiently investigated. This study recruited frontline Chinese police officers to address this research gap, for the first time separately examining the independent mediating roles of perceived social support and sleep disturbances, and their chained mediation effect linking police work stress to life satisfaction. Police work stress was significantly and negatively associated with life satisfaction. Moreover, work stress exerted significant negative indirect effects on life satisfaction through perceived social support and sleep disturbances, establishing a statistically robust serial mediation pathway. Crucially, these direct and indirect effects remained stable and significant after adjustment for key demographic covariates, thereby substantiating the reliability of the observed relationships.

### 4.1 Correlations between variables

Supporting our hypotheses, there were significant correlations among police work stress, life satisfaction, perceived social support, and sleep disturbances. Specifically, police work stress was significantly negatively correlated with life satisfaction, aligning with prior evidence from police cohorts demonstrating that elevated job stress coincides with diminished life satisfaction, subjective well-being, and overall quality of life (Alexopoulos et al., 2014). Using a sample of Indian police officers, Lambert et al. (2021) further documented a significant negative association between occupational stress and life satisfaction, implying that work stress spills over into individuals’ global life assessments. Similarly, in Ryu et al. (2020), police occupational stress predicted reduced subjective well-being, with coping strategies moderating this relationship. These findings align with our results, collectively suggesting that elevated occupational stress among police officers transcends mere workplace difficulties, manifesting as reduced life satisfaction.

In our correlation analyses, police work stress was significantly negatively correlated with perceived social support and significantly positively correlated with sleep disturbances. Previous studies identified perceived social support as a critical resource protecting police officers’ mental health: sufficient perceived social support correlates with superior psychological well-being and occupational adjustment (Campos et al., 2023; Hansson and Padyab, 2023; Padhy et al., 2023). Sleep impairment is highly prevalent among police officers, stemming largely from irregular night shifts, rotating rosters, frequent on-duty assignments, and mandatory rapid emergency response (Garbarino et al., 2019; Garbarino and Magnavita, 2019). Taken together, these findings reveal that elevated work stress among police officers corresponds to lower perceived social support and more severe sleep dysfunction.

This study also detected a significant positive correlation between perceived social support and life satisfaction, alongside significant negative correlations between sleep disturbances and both perceived social support and life satisfaction. These correlations align with earlier empirical findings (Angehrn et al., 2022; Chen et al., 2025), demonstrating that perceived social support and sleep deficits are two key explanatory mechanisms regarding the police occupational stress–life satisfaction relationship.

### 4.2 Chain mediation role of perceived social support and sleep disturbances

We identified a significant serial mediating effect of perceived social support and sleep disturbances in the police work stress–life satisfaction relationship. This finding demonstrates that police work stress not only directly predicts life satisfaction but might also indirectly predict it through the “perceived social support → sleep disturbances” chain path. Elevated occupational stress predisposes police officers to feel they lack social support; this perception further exacerbates sleep disturbances, culminating in reduced life satisfaction. This serial mediation effect corroborates research establishing that occupational stress, social support, and sleep quality jointly shape police officers’ psychological well-being (Angehrn et al., 2022; Chen et al., 2025).

This sequential mediating mechanism can be explained in two ways. First, the COR theory posits that chronic stress depletes individuals’ psychological, emotional, and social resources, hindering people from proactively seeking assistance, sustaining functional interpersonal communication, or recognizing supportive feedback from family, coworkers, and institutional bodies (Hobfoll, 1989). Police officers face persistent, unpredictable stressors including law enforcement hazards, irregular shift work, emergency response duties, and public scrutiny. Prolonged exposure to such occupational strain renders police personnel more vulnerable to feelings of alienation and reduced access to social support. Consistently, social support buffers the detrimental impacts of occupational stress among police, whereas inadequate perceived social support amplifies stress responses (Cohen and Wills, 1985; Patterson, 2003).

Second, lower perceived social support is a salient risk factor for sleep disturbances. Adequate social support mitigates stress arousal by offering emotional comfort, instrumental problem-solving assistance, and a greater sense of safety. By contrast, police officers with inadequate perceived social support might experience heightened vigilance and repetitive rumination post-shift, impairing sleep initiation, continuity, and overall quality (Åkerstedt, 2006; de Grey et al., 2018). This interpretation is consistent with previous empirical investigations of police cohorts (Angehrn et al., 2022; Birhan et al., 2025; Garbarino et al., 2019). Accordingly, perceived social support is not merely an independent correlate of life satisfaction, but also a critical intermediate pathway translating occupational stress into sleep disturbances.

## 5 Implications

This study offers several theoretical implications for research on police occupational mental health. By verifying the serial mediating roles of perceived social support and sleep disturbances, our findings reveal the step-by-step psychological mechanism linking occupational stress to life satisfaction among Chinese front-line police officers. Rather than treating social support and sleep as isolated variables, this study extends existing stress theories and provides non-Western empirical evidence illustrating how work-related strain undermines well-being via the serial mediation of reduced perceived social support and sleep disturbances.

This research also yields meaningful managerial implications for police organizational governance and mental-health services. Interventions to boost officers’ life satisfaction should move past individual-level stress regulation toward systemic improvements in social support infrastructure and sleep-health management. Police departments are advised to develop multi-tiered support mechanisms and roll out regular sleep-health screenings alongside evidence-based intervention programs including mindfulness meditation, progressive muscle relaxation, Tibetan singing bowl therapy, and music imagery (Gaskin et al., 2026; Cai et al., 2025; Donato et al., 2026), delivering accessible mental-health resources for front-line officers.

## 6 Limitations

Several methodological caveats warrant consideration when interpreting our findings. First, although our SEM results support the proposed sequential mediation pathways linking occupational stress to life satisfaction via perceived social support and sleep disruption, the inherent correlational structure of the data—given the cross-sectional data collection—precludes definitive claims regarding temporal precedence or causal ordering. Longitudinal follow-up designs with cross-lagged panel models are recommended to disentangle temporal ordering and capture dynamic variations across study variables. A second concern is our reliance on self-reported measures for all study variables—an approach inherently vulnerable to socially desirable responding. Although we implemented procedural strategies during data collection to mitigate common-method bias, and conducted the Harman single-factor test and competing-model CFA as diagnostic checks, these statistical approaches can only indicate the absence of severe common-method bias rather than provide definitive proof of its non-existence. Police officers might still adjust their self-reports owing to professional identity constraints, confidentiality worries, or self-presentation effects. Subsequent research could integrate multi-source data, including semi-structured interviews, peer or family ratings, organizational administrative records, and wearable sensor measurements, to enhance reliability and ecological validity. Third, our findings are limited in generalizability. Data were solely gathered from Chinese police officers, whose institutional frameworks and incentive mechanisms differ from law enforcement systems adopted in other countries. Such institutional gaps may alter the magnitude of associations among work stress, social support, sleep, and life satisfaction. Thus, cross-national studies are required to verify our mediation model’s replicability. Fourth, police occupational role was not formally collected as a quantitative variable, precluding subgroup comparisons across police functional departments, despite the sample covering multiple divisions (criminal investigation, public security administration, traffic management, prison and correctional services, exit-entry administration, and others). Police rank was also not entered as a control variable: under China’s dual-track promotion system, rank is primarily seniority-based and highly correlated with length of service, so police tenure was included instead to capture rank-related variance while avoiding multicollinearity. Future research should measure both variables to examine subgroup differences and further validate model robustness.

## 7 Conclusion

This study examined the relationships among police work stress, life satisfaction, perceived social support, and sleep disturbances in Chinese on-duty police officers and explored the chained mediating effect of perceived social support and sleep disturbances on the police work stress–life satisfaction relationship. There were significant correlations among all four variables. Police work stress not only directly predicted life satisfaction, but also indirectly predicted it via perceived social support and sleep disturbances. In addition, perceived social support and sleep disturbances exerted a significant chained mediating effect in the relationship between work stress and life satisfaction. This finding suggests that work stress could reduce police officers’ perceived social support, worsen sleep disturbances, and thus reduce life satisfaction. In summary, this study identified multiple pathways through which police occupational stress contributes to reduced life satisfaction. For police mental health services and organizational management, comprehensive interventions to strengthen social support systems and enhance the recognition and use of social support are recommended, in addition to evidence-based interventions to improve sleep quality, thereby improving police officers’ life satisfaction and overall adaptive functioning.

## Data Availability

The raw data supporting the conclusions of this article will be made available by the authors, without undue reservation.
