# Supplementary material for: Association between police work stress and life satisfaction: the chain mediating roles of perceived social support and sleep disturbances
Source: Front Psychol. 2026 Sep 17;17:1938729. doi: 10.3389/fpsyg.2026.1938729 (PMC13626913; doi:10.3389/fpsyg.2026.1938729)
Supplement: Supplementary file 1 [file Data_Sheet_1.PDF]

Supplementary materials

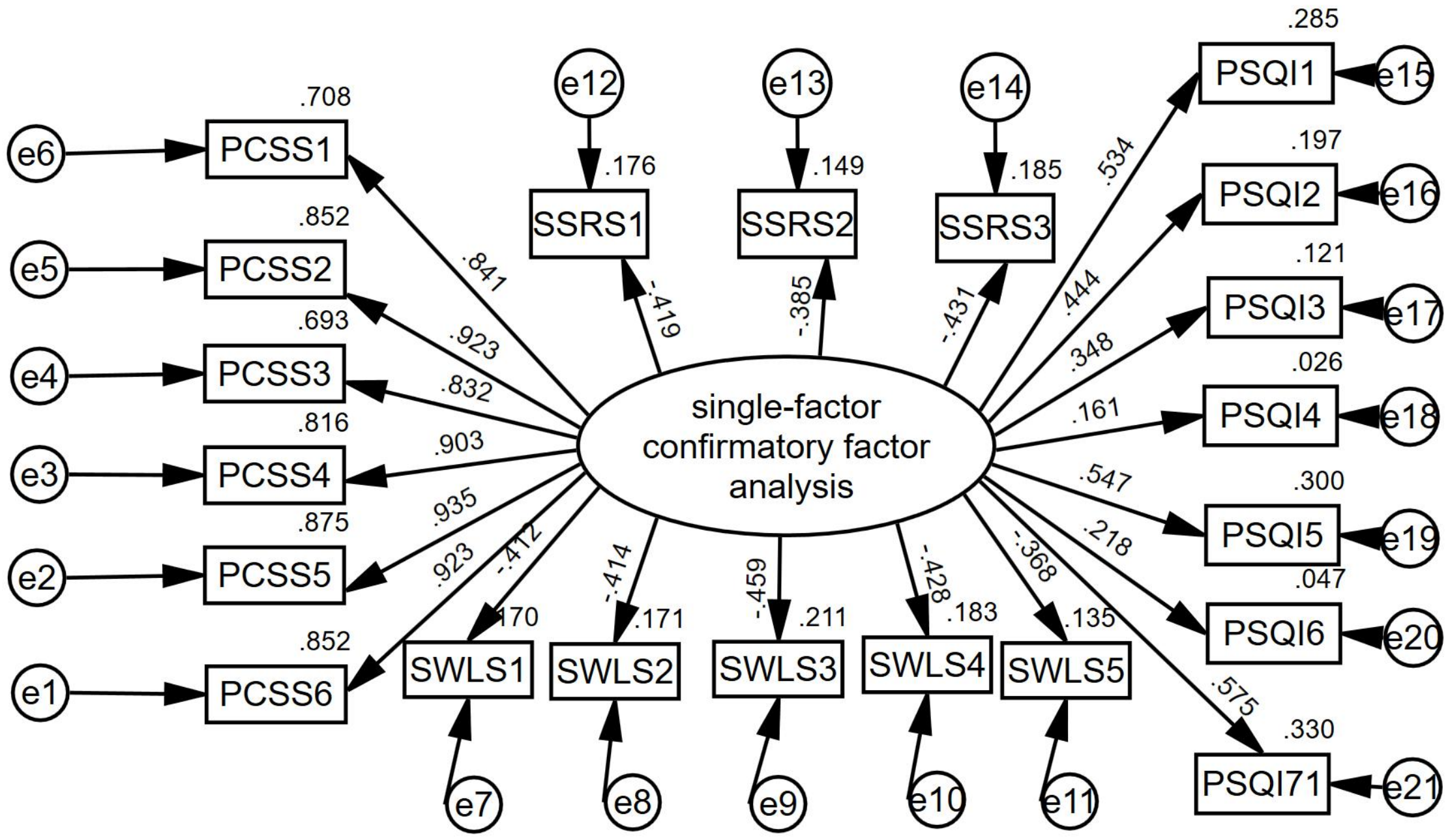

Figure S1. Single- factor confirmatory factor- analysis model for common- method bias assessment

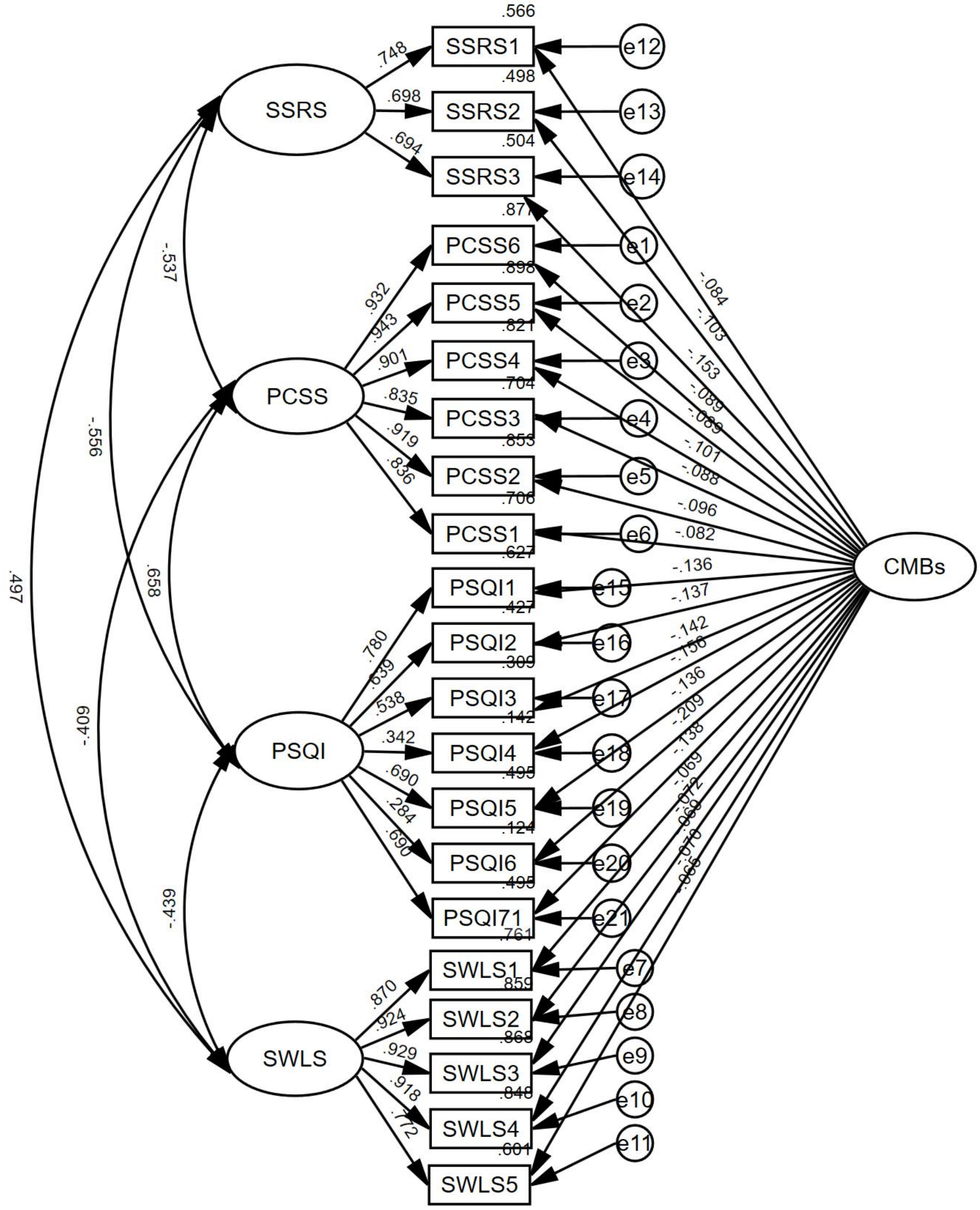

Figure S2. Four-factor confirmatory factor-analysis competing model for common-method bias assessment.

Table S1. Model-fit indices for the single-factor and four-factor confirmatory factor-analysis models assessing common-method bias.

Fit indices	Criteria	Single-factor model	Four-factor model
χ^2/df	$1 < \chi^2/df < 5$	29.811	4.884
RMSEA	< 0.08	0.190	0.070
SRMR	< 0.08	0.166	0.047
RMR	< 0.08	0.391	0.05
GFI	> 0.90	0.518	0.905
AGFI	> 0.90	0.410	0.88
NFI	> 0.90	0.577	0.933
RFI	> 0.90	0.530	0.923
IFI	> 0.90	0.585	0.946
TLI	> 0.90	0.538	0.938
CFI	> 0.90	0.585	0.946

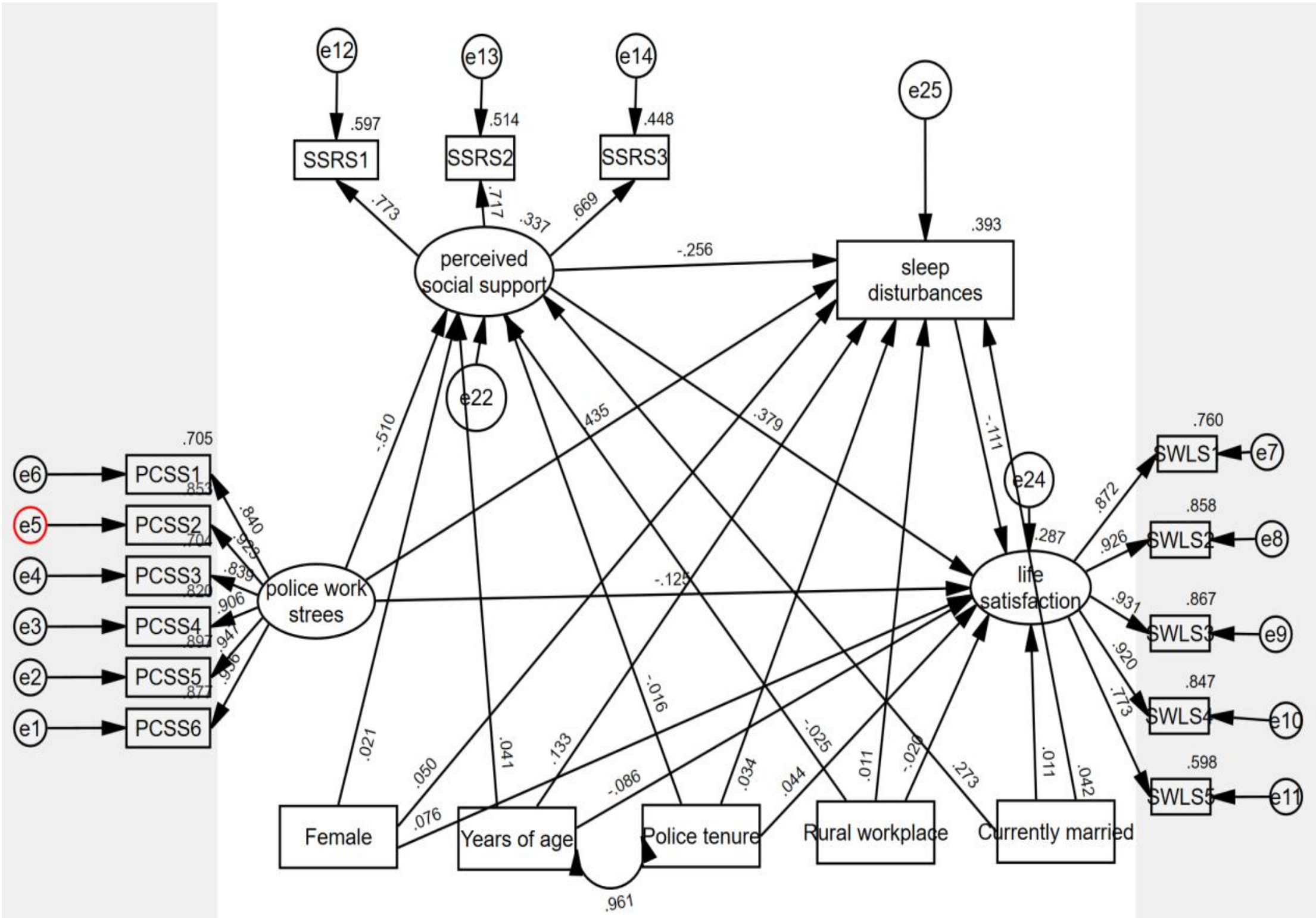

Figure S3. Covariate- adjusted structural equation model with demographic control variables (gender, age, police tenure, workplace location, and marital status).
